# Do ICU patients' discharge characteristics change with Swiss Diagnosis Related Groups?

**DOI:** 10.1186/cc12421

**Published:** 2013-03-19

**Authors:** G Rauchenstein-Ziltener, CA Rüst, P Fodor, S Blumenthal

**Affiliations:** 1 Tiemli City Hospital, Zurich, Switzerland

## Introduction

Swiss Diagnosis Related Groups (SwissDRG) have been effective since 1 January 2012. The influence of this new system on patients' discharge characteristics from a large ICU is not known. With the introduction of the DRG we expect patients to be discharged after a shorter length of stay on the ICU and with higher severity of illness.

## Methods

The ICU of the City Hospital Triemli in Zurich has an interdisciplinary organization with surgical and internal medical patients, with a maximum occupancy of 18 beds and a center function for the surrounding hospitals. In this ongoing prospective observational study, we collect and analyze the anonymized data of all patients discharged from our ICU prior to and after the introduction of the SwissDRG. The primary endpoint was the length of stay on the ICU in hours. The secondary endpoints were the severity of illness of the patients at the time of discharge, detected by the scoring system SAPS II as well as measured by the number of readmissions to the ICU. Initially all patients were analyzed and in a second step only patients within percentiles 6 to 94 were considered. We also analyzed the subgroups of patients referred internally, patients sent back to referring hospital and patients regionalized to a homebase hospital. The statistics have been done with SPSS and *P *0.05 was considered significant.

## Results

We present the results of an 18-month period, 9 months prior to and 9 months after the introduction of the SwissDRG. Data of 1,491 and 1,492 patients were analyzed, respectively. When all patients were included, we found prior to and after the introduction of the DRGs a comparable length of stay on the ICU (mean ± SD of 52.1 ± 2.2 hours vs. 50.82 ± 2.2 hours), no difference in the severity of illness at discharge detected by the SAPS II (mean ± SD of 27.9 ± 0.3 vs. 28.4 ± 0.3) and the number of readmissions (91 vs. 92). There was also no significant difference when only percentiles 6 to 94 were included or when the three subgroups were analyzed.

## Conclusion

Up to now, the introduction of the SwissDRG has no influence on patients' discharge characteristics from a large ICU. Data assessment will continue and further data analysis has to be performed.

